# Fluids and barriers of the CNS: a historical viewpoint

**DOI:** 10.1186/2045-8118-8-2

**Published:** 2011-01-18

**Authors:** Shane A Liddelow

**Affiliations:** 1 Department of Pharmacology, University of Melbourne, Australia

## Abstract

Tracing the exact origins of modern science can be a difficult but rewarding pursuit. It is possible for the astute reader to follow the background of any subject through the many important surviving texts from the classical and ancient world. While empirical investigations have been described by many since the time of Aristotle and scientific methods have been employed since the Middle Ages, the beginnings of modern science are generally accepted to have originated during the 'scientific revolution' of the 16^th ^and 17^th ^centuries in Europe. The scientific method is so fundamental to modern science that some philosophers consider earlier investigations as 'pre-science'. Notwithstanding this, the insight that can be gained from the study of the beginnings of a subject can prove important in the understanding of work more recently completed. As this journal undergoes an expansion in focus and nomenclature from cerebrospinal fluid (CSF) into all barriers of the central nervous system (CNS), this review traces the history of both the blood-CSF and blood-brain barriers from as early as it was possible to find references, to the time when modern concepts were established at the beginning of the 20^th ^century.

## Review

### The protective barriers of the brain

A large amount of information available on the barriers of the brain, especially in development, remains a tangled and somewhat controversial matter, despite research in the field going back centuries. This is partly due to the misunderstanding of several of the early experiments conducted by German neuroanatomists (such as Ehrlich and Goldmann), but also because there is a common belief that the barriers of the developing brain are immature. Some of these arguments are teleological at best, with Barcroft [1] arguing that:

'There is no reason why the brain of the embryo should require an environment of very great chemical constancy. It will of course require a certain minimum of the various materials necessary for growth, but otherwise on first principles we must suppose that the good things of life may exist in and may vary in the foetal blood to an extent much greater than in the neonatal.'

**Figure 1:**
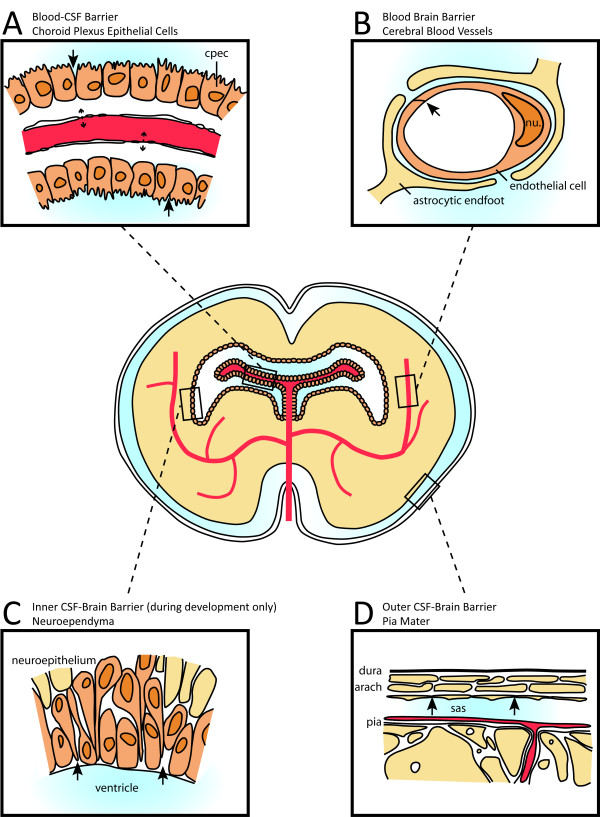
**Illustration of the sites of brain barriers in the developing and adult brain**. **A. **The blood-CSF barrier. A barrier between choroid plexus blood vessels and the CSF. The blood vessels in the choroid plexus are fenestrated and form a non-restrictive barrier (dotted arrows). The choroid plexus epithelial cells (cpecs) are joined by functional tight junctions towards their apical surface that stop the movement of molecules (arrows). **B**. The blood-brain barrier. A barrier between the lumen of cerebral blood vessels and the brain parenchyma. The endothelial cells have luminal tight junctions (arrow) that form the physical barrier stopping the movement of molecules out of the vasculature. Astrocytic endfeet are in close association of the cerebral blood vessels and form what is known as the 'neurovascular unit'. The endfeet are not necessary for blood-brain barrier integrity. **C**. The inner CSF-brain barrier, present only during early development. A barrier between the CSF and the brain parenchyma. The neuroependymal cells lining the ventricular wall (orange) are connected by 'strap junctions' [2], halting the exchange of large molecules such as proteins between the CSF and brain (arrows), but not of smaller molecules like sucrose. This barrier is not present in the adult brain due to a loss of strap junctions. There is no restriction of movement at this time. **D**. The outer CSF-brain barrier. A barrier between the CSF-filled subarachnoid space (sas) and overlying structures. The blood vessels in this area are fenestrated and provide little by way of a barrier, but the outer cells of the arachnoid membrane (arach) are connected by tight junctions. Abbreviations: arach, arachnoid membrane; cpec, choroid plexus epithelial cells; dura, dura mater; nu., nucleus; pia, pia mater; sas, subarachnoid space. Adapted with permission from [4].

This misconception is amplified by the term 'blood-brain barrier' incorrectly used to describe all anatomical barriers of the brain, with no clear specification as to which particular barrier a researcher is considering. There are four (major) independent barriers in the brain (Figure 1). These are:

1. The blood-CSF barrier, at the level of the choroid plexus epithelial cells (Figure 1A).

2. The blood-brain barrier at the level of the endothelium of cerebral blood vessels (Figure 1B).

3. The CSF-brain barrier created by separation of the ventricular system from the extracellular fluid of the brain, which is only present in the embryo [2] Figure 1C.

4. The arachnoid barrier between the CSF in the subarachnoid space and the dura mater and overlying tissues (Figure 1D).

The most closely scrutinised is the blood-brain barrier, in which the vascular endothelial cells of the central nervous system are connected by tight junctions, forming a restrictive barrier to the movement of molecules and electrolytes between the brain and the blood. It was originally thought that astrocytes were required for induction of the barrier capabilities of these cells postnatally [3], however more recent work has shown this to be untrue [4,5]. It is now believed that pericytes are important for the induction of the barrier during embryogenesis, during a period when astrocytes are not present in the central nervous system [5].

In addition to these four barriers, there are studies describing the importance of the arachnoid barrier (at the level of the cerebral blood vessels on the outer surface of the brain in the arachnoid [4]), the blood-retinal barrier (the retina being made of nerve endings, and as such comprising the brain [6-9]) and the nose-brain barrier (at the level of the olfactory receptors, also neurons, in the nose [10]). Each of these barriers is functionally tight to small molecules, due to the presence of junctions: either tight junctions in the case of the blood-brain and blood-CSF barriers, or 'strap' junctions [2] in the case of the two (the inner CSF-brain barrier at the level of the ventricular wall, and the outer CSF-brain barrier on the outer surface of the cortex) CSF brain barriers present in the embryo. In the case of the blood-retinal barrier, it has been shown that an analogous barrier is present separating the blood from the retina, which remains unstained after an intravenous injection of trypan blue, but stained after injection into the vitreous humour [11]. In addition, Goldmann viewed the choroid plexus as being analogous to the placenta, and indeed he showed that trypan blue was unable to pass from the maternal to fetal circulation of a pregnant dog [12].

As well as the work completed by Stern using toxins (see below), the use of lipid-soluble narcotics, which have a far more rapid action on the central nervous system, began to show that lipid-solubility played a large role in the penetration of molecules across brain barriers [13]. From this time onwards, the appearance of better analytical techniques meant that the use of basic dyes (as used by Ehrlich, Goldmann and Lewandowsky, among others) could be discarded. This saw an increase in the number of available compounds used in experiments; for example, the newly developed radioisotopes meant that tracer levels could be measured in a quantitative way. The seminal work by Hugh Davson using a number of small molecules, electrolytes and non-electrolytes, confirmed that the lipid solubility of a substance was one of the main determinants for its penetration into the brain (for review see [14]). These 'modern' concepts of the brain barriers will not be further covered here as they form the main focus of another article in this journal [15]. This review will instead concentrate on the historiography of the system, ending around the turn of the 20^th ^Century.

#### The cerebrospinal fluid

It is now well documented that the CSF is the nutrient-rich fluid that bathes the brain and spinal cord as well as filling the ventricles of the brain and spinal canal. The composition of the CSF is tightly controlled, such that substances including amino acids, vitamins, minerals, ions and proteins are held at very specific concentrations depending on stage of development [16]. The actual 'discovery' of the CSF is marred by poor record keeping requiring a treasure-hunt of sorts to trace its exact origins. The beginnings of the CSF story are found in the writings of the ancient Egyptians, with the earliest reference and description of fluid contained in the brain identified on the Edwin Smith Surgical Papyrus [17]. This document, with a proposed date of 1700BC (although dealing with material from a thousand years earlier), describes several ailments of injured persons. In relation to the brain and the meninges the papyrus comments on a patient with a skull fracture that exposed the brain:

'... it is a big fracture, which is open to the inside of his skull and the membrane that covers his brain; it has fractured and a liquid gushes from inside his head.' (Edwin Smith Surgical Papyrus, 1700BC, from the National Library of Medicine, Bethesda, MD, USA).

About a thousand years later in ancient Greece, Hippocrates (129 - 219AD [18]) described hydrocephalus in both domestic animals and humans and knew that it was the result of 'water' inside the head. The ancient descriptions of the CSF including those by Cladius Galenus, known simply as Galen [19], reported cases of CSF leak, or rhinorrhea, when patients had a spontaneous eruption of fluid from the nasal cavity [20]. Following this observation, Galen proposed that CSF was released into the nose from the pituitary and ethmoid regions of the brains of sick individuals. Galen's fluid, the 'psychic pneuma' was made by the brain and transported by the nerves to peripheral organs [21]. The general belief in Greece at the time was that the body and spirit were tempered by vapours that were transported throughout the organ systems. Indeed Galen's description of two proposed fluids of the brain stated:

'... there are two kinds of these residues, one vaporous and fuliginous, tending naturally to pass upward, the other watery and slimy so to speak which sinks down of its own weight.' [22].

**Figure 2:**
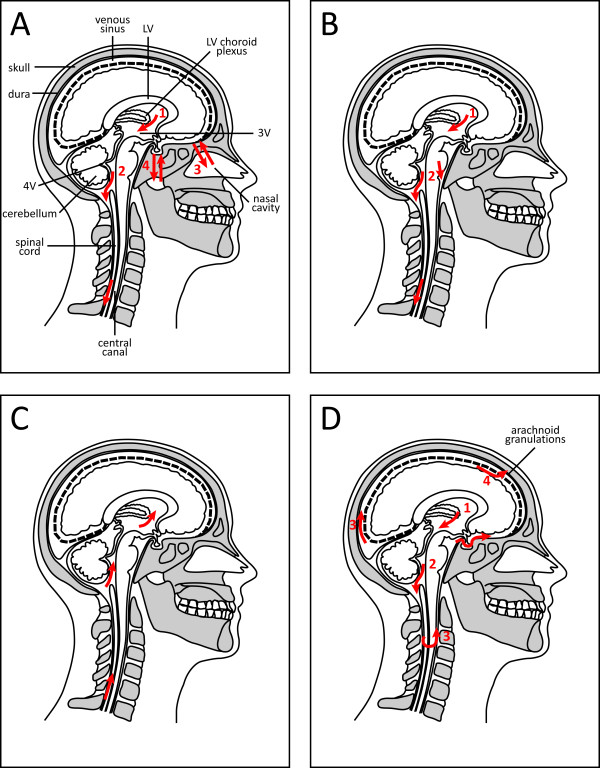
**The evolution of our understanding of CSF production and pathway in relation to brain spaces**. **A. **Cladius Galenus' (Galen, 129 - 219AD) concept of the CSF pathway. In his writings Galen describes a lateral ventricular choroidal origin (1) and exit through the fourth ventricle to the spinal canal (2). He also erroneously describes movement of the fluid across the cribriform plate into the nasal cavity (3) and across the infundibulum to the palate (4). **B**. Albrecht von Haller's (1708 - 1777AD) CSF pathway correctly stated the origin of CSF from the ventricles (1), with exit from the fourth ventricle (2) and down the spinal canal for venous absorption. This early description was essentially correct. **C**. François Magendie's (1783 - 1855AD) concept of the CSF pathway was exactly opposite to the system of both von Haller and Galen. **D**. The modern description of the CSF pathway. CSF is produced by the choroid plexuses (1) from where it moves from the lateral ventricles into the third and fourth ventricles (2). It then flows across the surface of the brain (3) and down the spinal canal (moving from the back to front (3) of the canal). CSF is then reabsorbed by the arachnoid granulations (4) back into the blood stream. The arachnoid villi are projections from the arachnoid layer of the meninges that connect with veins via the venous sinus. Absorption into lymphatics also occurs (not shown). Abbreviations: 3V, third ventricle; 4V, fourth ventricle; LV, lateral ventricle.

Neither Galen nor his contemporaries were able to properly describe the fluid, through no failure in their anatomical or descriptive skills, but due to the nature of the autopsies: at this time it was common to sever the head of bodies before embarking on the post-mortem examination and many bodies were hung to bleed before dissection which would begin to enable clearer visualization of the organs. Such an act would cause a loss of CSF, thus the ventricles would only have been viewed in a drained state. Despite these shortcomings, Galen was able to propose a concept of CSF production and movement not very different from that described today. He proposed that CSF was produced by the lateral ventricles in the brain when a patient was sick, and passed from the posterior ventricle into the subarachnoid space. From here, the fluid was suggested to be drained by two means, firstly via the cribriform plate at the top of the nasal cavity and thus directly out the nose, and secondly through a network of tubes near the infundibulum and out through the hard palate at the back of the throat (Figure 2).

Well over a thousand years later Andreas Vesalius (1514 - 1564 [23]) at the University of Paris began to demonstrate errors in Galen's descriptions of human anatomy:

'How many things have been accepted on the word of Galen ... and often contrary to reason. [...] Indeed, I myself am wholly astonished at my stupidity and too great trust in the writings of Galen and other anatomists.' [23].

**Figure 3:**
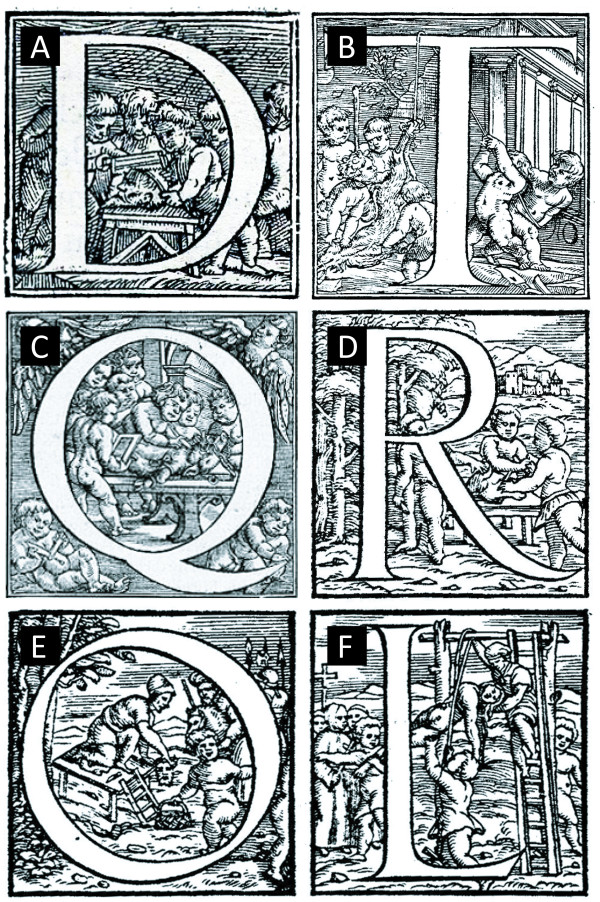
**Decorated letters from Vesalius' *De Humani Coporis Fabrica *(1543)**. **A**. The act of removing the head of cadavers prior to dissection can be seen, as cherubs set to the head of a man with a bone saw. **B**. Once decapitated, the subjects (human or beast) were hung to drain the blood from the body. This enabled a much 'cleaner' visualisation of the internal organs. **C/D**. At the same time as public dissections of human subjects were occurring, the dissections of beasts was also conducted. This increased the availability of subjects, but also allowed a better comparison to the works of the ancients who worked mostly on animals. In **C**, the cherubs can be seen dissecting a boar, while another looks on, reading from a book (possibly Galen's). **E/F. **The difficulty in obtaining human subjects was such that at the time of Vesalius' bodies were often obtained via grave robbing (**E**) or by taking the bodies of executed criminals (**F**). All images are reproduced by gracious permission of Her Majesty The Queen, from the Royal Collection ^© ^2010, Her Majesty Queen Elizabeth II.

Vesalius improved on the knowledge of the CSF, as he considered that the fluid (rather than the 'vapour') was the major component of the ventricular system, even of healthy individuals. Although a greater understanding of the system was made at this time, Vesalius still believed the notion that the fluid was excreted through the pituitary and cribriform plate. In addition, the historical idea that the CSF was 'directed through the passages of the nerves, like the vital spirit [blood] through the arteries' [23] was still believed. There were several problems with the collation of information on the human form at this time - the most prominent being the lack of available cadavers for dissection. Vesalius hints at the way around these problems in the decorated text in his 1543 text '*De Humani Corporis Fabrica*' - grave robbing, taking corpses from public execution, the removal of newborns from sick or unconscious mothers, as well as the use of animals in addition to human subjects (Figure 3).

**Figure 4:**
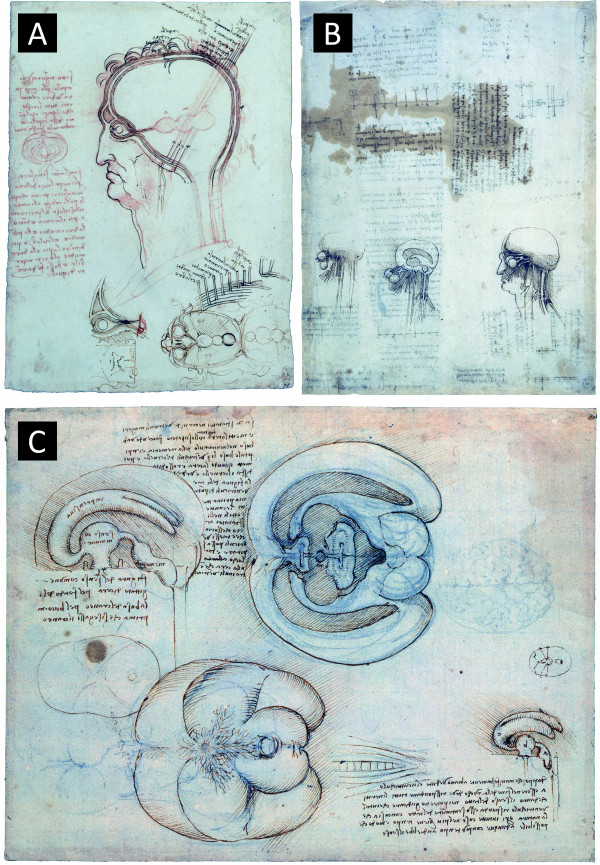
**Sketches by Leonardo da Vinci on the anatomy of the brain**. **A**. The layers of the scalp compared to an onion (1489). The earliest drawings by da Vinci on the ventricles of the brain show them to be connected to the eye and moving backwards, into the brain. In this drawing he also likens the meninges of the brain to the layers of an onion (left hand side of image). **B**. Studies of the eyes and brain (1508). Later studies by da Vinci on the neuroanatomy of man display a better understanding of the ventricles of the brain and of nerves permeating to peripheral areas. This increase in understanding is likely due to the use of wax casts made of the ventricular system of other 'lower' animals, such as the ox (see **C**). **C**. The cerebral ventricles of the brain of an ox (unknown date: 1508 - 1510). Here da Vinci describes the methodology of injecting warmed wax into the ventricular system of the ox, allowing it to cool, then visualising and sketching the mould that is made. Though not describing explicitly the use of bovine species, we can assume that da Vinci has by the presence of the bovine equivalent of the circle of Willis, the *rete mirabilis*, in the lower image. All images are reproduced by gracious permission of Her Majesty The Queen, from the Royal Collection ^© ^2010, Her Majesty Queen Elizabeth II.

At the time when Vesalius was producing his intricately detailed images of the human form, Leonardo da Vinci was also producing sketches on and about the form of the human body. Of most interest to this narrative are his depictions of the ventricular system of man. In a collection of three drawings from 1489, held in the Royal Collection of Her Majesty Queen Elizabeth II (Figure 4), da Vinci shows his original concept of the ventricular system as three consecutive spheres behind the eye (a holy trinity, based as much on religious teaching as scientific knowledge). In this same image da Vinci hints at the presence of the meninges of the brain, likening the coverings of the skull to the layers of an onion (Figure 4A). Later images, from 1508, show the influence that the production of wax casts on the ventricles had on da Vinci's understanding of their positioning. Unfortunately however, for all the correctness that was revealed by the use of wax casts, the diagrams of da Vinci were not readily available for several centuries after his death due to the cryptic way in which he wrote in his notebooks [24].

Later, investigators at Oxford University shed new light on the problem, with William Harvey (1578 - 1657 [25]), although never working directly on the ventricular or nervous systems, correctly describing the detail of the systemic circulation and properties of the blood being pumped around the body by the heart [25]. This return of the blood by the venous system allowed the depiction of a new outlet for fluids from the brain. Thomas Willis (1621 - 1675 [26]) used this new-found information provided by Harvey to completely re-evaluate brain anatomy in his opus '*Cerebri Anatome*' (1664). In this work, Willis was the first to correctly forecast the choroid plexus as the source (by secretion) of the CSF, however, he still held to the belief that CSF passed into the nose across the cribriform plate.

Following another century with no real improvement to the description of the presence of CSF in the normal healthy human nor of its movement through the ventricles, an independently working Swiss scientist, Albrecht von Haller (1708 - 1777 [27]), described CSF circulation in the mid 1700s.

' ... the vapour, which is secreted into the ventricles of a healthy person, is in like proportion absorbed again by the inhaling veins and that if there be any excess it descends through the bottom of the ventricles to the basis of the skull and into the loose cavity of the spinal marrow.' (Haller, 1747; as quoted by [28]; see also Figure 2B for review).

At this stage, a large body of knowledge was starting to emerge, not only of the structure of the ventricles but also of the presence of the CSF within these ventricles and on the outer surface of the brain and the spinal cord. It is interesting to find most historians of neuroanatomy speaking of the 'discovery' of the CSF by François Magendie (1783 - 1855 [29]). Magendie was adamant that any progress in the health sciences could not be made without the most ardent of vivisections. His extensive use of the technique and arrogance in arguing the reasons for animal experimentation drew much criticism from many of his contemporaries and later scientists such as Charles Darwin [30,31]. Magendie appeared to have been aware of the descriptions of the CSF by preceding scientists, However, he preferred to ignore or plainly dispute their contributions, even if he himself was incorrect. In a lecture published by the Lancet in 1836 Magendie comments:

'The existence of the cerebro-spinal fluid was long ago remarked by the ancient writers on medicine; but these observations were either lost or remained unnoticed [...] you will find it mentioned now for the first time, and then in consequence of the experiments I had made upon the subject' [32].

He then goes on to claim:

'I was the first to determine its [CSF] existence by direct experimentation' [33].

Indeed, although describing CSF and its flow through the ventricular system, Magendie was erroneous in his understanding of direction of its movement (Figure 2C). The outcomes of these misconceptions by Magendie, although brief, were enduring in their effect. Such 'new findings' that occurred after Magendie's lectures of the early 19^th ^century included Faivre's description of the production of CSF by the choroid plexus some 200 years after Thomas Willis.

The direction of flow of the CSF, outwards from the cerebral ventricles, was again re-established by Key and Retzius and has remained the accepted description until modern times [21]; see also Figure 2D). The notion that the CSF was not simply a filtrate of plasma was outlined in the thesis of William Mestrezat [34]; however, the actual production/secretion of CSF by the choroid plexus was proved indisputably by Walter Dandy (1886 - 1946 [35]) when he plugged one foramen of Monro in the brain of a kitten and caused hydrocephalus of the ipsilateral ventricle. This elegant experiment showed that cerebrospinal fluid had an intraventricular source. Further to this experiment, Dandy blocked the foramen of Monro in one brain while simultaneously removing the choroid plexus on the same side. In this experiment, there was no hydrocephalic response [36]. These paired experiments showed unequivocally that the CSF arises in the ventricles and that the choroid plexus must be the site of production of the CSF.

Far from being a passive filtrate of the plasma, it has been shown that a certain amount of metabolic work is required for the formation of CSF from the blood, and the hydrostatic pressure of the capillaries of the choroid plexus are insufficient to supply this energy alone [36]. This brought about three assumptions regarding the production of the CSF by the choroid plexus epithelial cells:

1. The actual structure of the choroid plexus epithelium itself, with high vascularisation and increased size of the plexus capillaries (up to 15 μm) compared with other brain capillaries (approximately 2 μm [37,38]) suggests cellular activity.

2. The chemical composition of the CSF indicates that it cannot be produced by simple ultrafiltration or dialysis alone [36,37,39].

3. Determination of the metabolic activity of the choroid plexus showed it to be very intense and of the same order as the liver and the kidney [37].

These observations, although attacked somewhat regularly in the mid-to-late 20^th ^century, have held firm, and the choroid plexuses are now known to be the principal site of production of CSF, by secretion [16].

#### The choroid plexus

Aristotle (384 - 322BC [40]), one of the students of Plato, at the age of 39, was requested by Phillip II of Macedon to undertake the education of his son Alexander [41]. It was during this period that he was believed to have composed several works on anatomy, which are unfortunately now lost. It was during Alexander's military exploits through Asia that Aristotle was able to obtain the means of extending his knowledge of the evolution and structure of the animals in the world in a more accurate way than was previously possible. It is stated that close to 1000 aides and numerous assistants in Greece and Asia were involved in the facilitation of his research that aimed to compose a system of zoological knowledge [41]. However, it has been noted that in a number of instances his trust in the testimony of others led him to make errors in description that personal observation might have enabled him to avoid. In total fourteen books were penned, constituting the extent of the Aristotelian Anatomy. Ten involved the History of Animals ('Πεζι Ζωων Ίστοζως'), while an additional four looked more closely at the Parts of Animals ('Πεζι Ζωων Μοζιων'). It was in the region of the brain that Aristotle made the most significant steps in anatomy, with his corrections of previous descriptions (by Polybus, Syennesis and Diogenes), which stated that all blood vessels originated from the brain [42]. Aristotle demonstrated that they arose from the heart, with their terminals occurring in the brain. He also correctly described the greater proportional size of the brain in man than in any other animal. However, errors arose when he stated that the organ was sparingly supplied with blood [41,43].

Aristotle was followed by Diocles of Carystus (4^th ^century BC [41]) and Praxagoras of Cos (4^th ^century BC [44]). Neither offered much new in the way of comparative anatomy, though they did rectify small errors of Aristotle's, such as accurately describing arteries and veins. It was also Praxagoras who is first regarded as properly describing both the brain and spinal cord as being neuronal in their makeup [43]. Later, one of Aristotle's pupils, Erasistratus (304 - 250BC [45]), distinguished himself from his teacher by dissecting a great number of human bodies (in addition to the bodies of 'lower' animals). It was now that the presence of more than one type of neuron was distinguished - but they were divided only into those involved in sensation and those involved in motion.

As the Hellenistic period (323 - 146BC) was ending due to the Roman conquest, the Greek language, adopted by many in the Asia region, was in decline. During this time, many who spoke Greek made their way to Rome. As the Romans were not distinguished for cultivating science, these Grecian scholars were able to employ their learning. Of all of the authors of the time, only one has claimed the title through history as 'The Anatomist' - Galen, often called the 'Physician of Pergamus'[19]. By age 28, Galen regarded himself as being in possession of all the knowledge obtainable through his teachers, causing him to leave Alexandria to offer himself as physician to Emperor Commodus [46]. Galen was a prolific writer and public lecturer; his nine book collection, the '*Anatomical Administrations*', was widely regarded as the most comprehensive anatomical description of the day. The writings of Galen stayed in anatomical and medical teachings for well over a millennium. In fact it was not until Andreas Vesalius published a printed description and illustrations of human dissections in 1543 that Galenic theory was equalled. Galen performed many daring operations, including brain and eye surgery, which were illegal in Rome at the time. In addition to proving that arteries of a living animal contain blood and not air (see [47]), he was the author that proposed the principle that the brain is the origin of the nerves for sensation and the spinal cord the origin of nerves for motion [48]. His neuroanatomical descriptions, although mostly gained from the dissection of lower vertebrates, are highly accurate - he properly described during dissection several protective layers on the outside of the brain, a complete view of all four ventricles (two lateral, third and fourth), the *velum interpositum*, the surface of the ventricles, and the choroid plexus. Galen refers to the structure thus:

'You will see in the ventricles what is called the 'choroid plexuses' (CHOROEIDÉ PLEGMATA). The followers of Herophilis call it a 'chorioid concatenation' (CHOROEIDÉ SUSTREMMATA), of course taking the name from the outer membrane of the foetus. It is a plexus of veins and arteries held together by delicate membranes.' [48]

The death of Galen, at age 90 (219AD [19]), coupled with the unsettled state of society in the later stages of the Roman Empire, saw the beginning of the downfall of ancient anatomy. Indeed the introduction of Christianity appeared to have a pernicious influence on the progress of science. During this age, the art of healing was dominated by ecclesiastics and superstitious beliefs, causing the art of anatomy to be mostly neglected [49]. It is therefore no wonder that there are scarcely any anatomical celebrities or medical diversity in the long period in Europe known as the Middle Ages. It is also fruitless to turn to Arabian physicians during this period for more hope, as their body of literature was more interested in the knowledge of natural history - the virtues of plants and alchemy. Indeed, it is the Koran that denounces unclean any person who touches a corpse, either human or animal (for a full review see [50]).

The ignorance of some Europeans and the burgeoning love of knowledge acquisition by the Italians saw a small revival of neuroanatomy in the early 1300s. Though commendable considering the clime of the day, their erroneous discoveries were unquestionably surpassed by Galen a millennium before. Mondino de Luzzi (1270 - 1326) demonstrated parts of the human body by dissecting female subjects in 1315 [51]. In his account of the cerebral membranes, he identified only the *dura mater*, and erroneously described only two ventricles (lateral and third). His short description of the choroid plexus is also inferior to Galen's, with the simple comment that:

'... the ventricles contain a blood-red substance, like a long worm.' [50]

Notwithstanding his gross misrepresentations, de Luzzi's descriptions of the human body formed the basis for anatomical texts for the next century.

After that brief period, the neurosciences were once more dormant for centuries until in 1502, James Berenger of Carpi, professor of anatomy and surgery at the University of Bologna, described the makeup of the body of deceased pigs [51]. His account of the brain is solid. He notes the presence of several ventricles, the segmentation of the brain's neuronal tissue, and finally, proves his sagacity by perceiving that the choroid plexus is 'comprised of veins and arteries' [50]. It was also Berenger who described the connections between each of the individual ventricles. During this golden age of anatomy in Italy, the French and English anatomists were still pre-occupied with the prejudices against the dissection of the human body. Indeed, even though the dissection of human subjects was approved, the use of dogs was preferred. But Dubois, for example, preferred to obtain his knowledge of the human form from an ardent admiration of Galen's texts [50].

In the mid 1500s, Charles Éstienne (1504 - 1564; [52]), although with a poorer understanding of the brain than those before him, correctly described the canal through the spinal cord, commenting on its connection with the ventricles of the brain. Owing however to the social and religious persecutions of the time, Éstienne was poorly credited with his findings and spent the last years before his death in 1564 in a dungeon. There was little addition to the greater understanding on the choroid plexus, or even the brain, in Europe until around 1664 when Thomas Willis (1621 - 1675 [26]) suggested that the choroid plexus contained structures that produced the fluid found within the ventricles of the brain (see above and [53]). Many had already assumed that the choroid plexus was integral to the production and secretion of the CSF, but it was the findings of Willis that suggested that the formation of this fluid was related to the juxtaposition between the choroid plexus and the pineal gland. A year later, in 1665, in '*De Anima Brutorum*', it was proposed that the choroid plexus was responsible for the absorption of CSF [50]. Following the work during the mid to late 1600s, there was an interlude in choroid plexus research until Peter Tarin (1725 - 1761 [54]) published his work '*Adversaria Anatomica*' in 1750 in which he described the surface of the choroid plexus as

'... a vascular fringe extending obliquely across the floor of the lateral ventricle, and sinking into the middle ...'

It was Tarin who commented that the two choroid plexuses of the lateral ventricles were connected through the foramen of Monro. The final piece of the choroid plexus/ventricular system puzzle arrived near the end of the 17^th ^century when Humphrey Ridley (1653 - 1708 [55]) described the presence of the choroid plexus in the third ventricle [56].

In present time, the choroid plexuses are recognised as highly vascularised tissues suspended in each of the cerebral ventricles (the two lateral, third and fourth). These specialised organs have many functions:

1. They form a physical barrier to the free diffusion of hydrophilic molecules.

2. They act as an enzymatic barrier for many substances derived from either the brain (via the CSF) or the blood.

3. They act as the site of other specific and non-specific transfer mechanisms, such as those for vitamins, minerals, growth factors or drug efflux, thus regulating the tightly controlled composition of the CSF.

4. They produce and secrete CSF that fills the ventricles and the subarachnoid spaces.

5. They synthesise some proteins (e.g. transthyretin) and growth factors.

In carrying out these precise functions, the choroid plexuses are simple in structure but complex in function. They comprise a central stroma with numerous blood vessels covered by a single layer of specialised and polarized ependymal cells (the choroid plexus epithelium) resting on a thick basement membrane. The choroid plexuses are part of the circumventricular organs and, as such, the vessels in the choroid plexuses are fenestrated from the earliest developmental stages and the tight junctional strands linking adjacent endothelial cells are discontinuous [57]. Accordingly, tracer molecules injected into the systemic circulation readily move out of the choroid plexus vessels and enter the connective tissue. The plexus epithelial cells are presented with a large volume of blood on their basal surface and bathed in CSF on their apical side - the surface of which is covered in many villi and cilia [58,59].

The protective diffusion barrier at the choroid plexus is provided by the presence of tight junctions between adjacent choroid plexus epithelial cells [59-67]. These junctions are characterized by an increase in the density of juxtaluminal lateral membranes [68]. Within the junction, the cell membranes of neighbouring cells are in close contact with each other at several places, forming what is known as *zonulae occludentes *[59,62]. The tight junctions prevent free movement of lipid insoluble molecules between the blood and CSF. Although the tight junctions between intimately apposed choroid plexus epithelial cells are present from very early in development, the selective movement of molecules across the interface is still possible. Becker and colleagues [69] showed that the plant enzyme horseradish peroxidase (HRP) enters the choroid plexus epithelial cells within coated vesicles. As choroid plexus epithelial cells contain a large number of mitochondria and show extensive Golgi complexes and endoplasmic reticulum, which appear to traverse from basal to apical membranes [70,71], it is now proposed that HRP (and other markers and endogenous molecules) translocate through plexus cells via a transcellular pathway utilizing these networks [71].

The tight junctions between choroidal epithelial cells, which resemble the *zonulae occludentes *in the brain endothelium [59] have been shown to be present in a number of species very early in development, including rat [67], mouse [72], sheep [63,65], chick [61,64], human [62,66,73] and the marsupial *Monodelphis domestica *[59]. Studies in *Monodelphis *have shown that these junctions are functionally tight to large molecules such as protein and to small tracers down to 286 Da [59]. It has been suggested [74-79] that there is a change in the tight junctional complex during development, with this change generally perceived as a maturation process of the junctions and hence of the brain barriers they represent. Ek *et al*. [80] states that if this were to be the case, there should be a corresponding change in barrier permeability, however in studies looking at these factors, the correlation between junction structures and barrier permeability is poor [63,78,81,82]. It is now accepted that the paracellular pathway across the blood-CSF barrier is occluded by tight junctions from the earliest stage of plexus development. The perceived change in permeability is due to other factors such as changes in the ventricular volume, transcellular transfer and CSF flow [83].

#### Barriers of the brain

**Figure 5:**
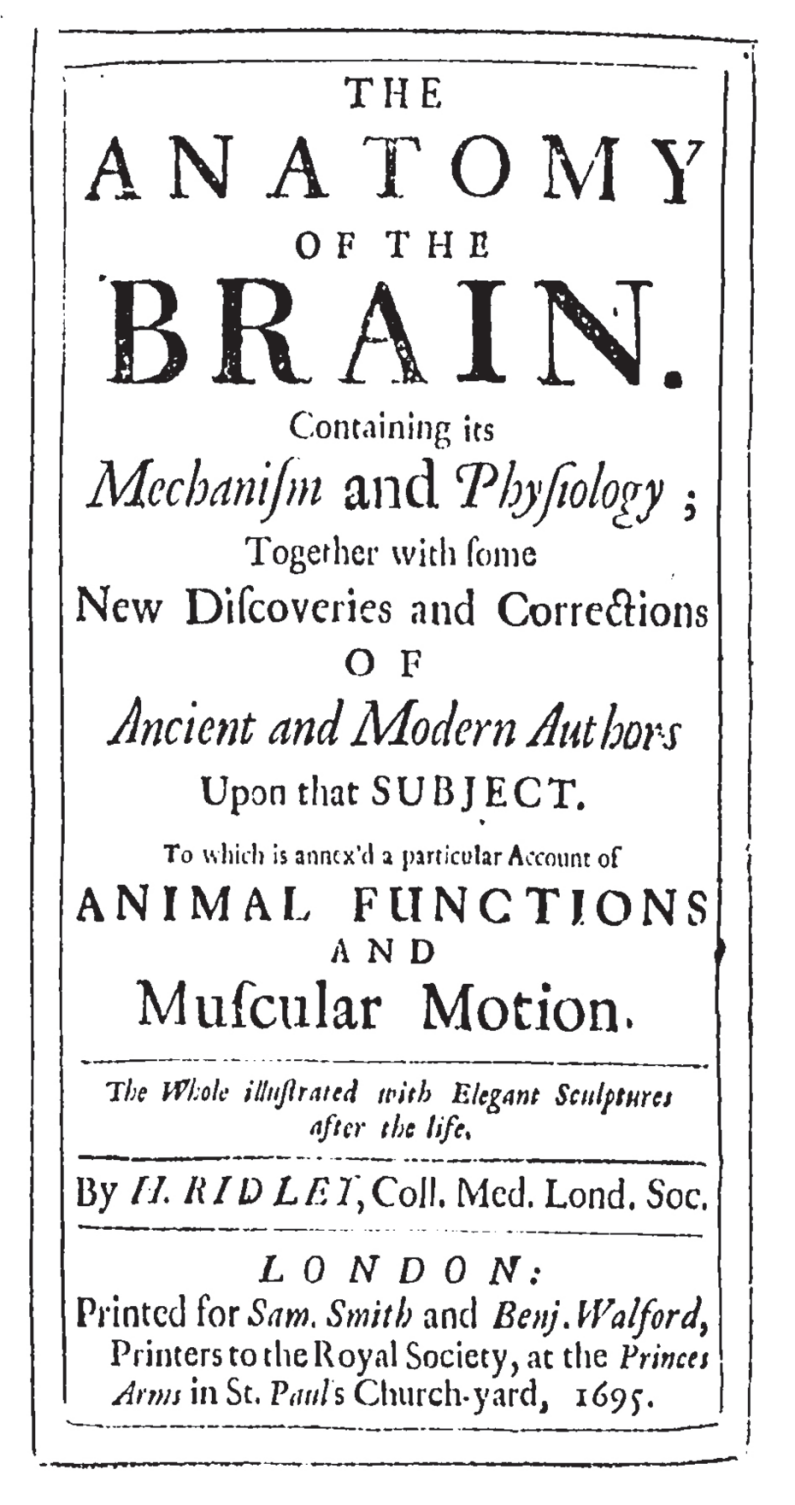
**Recto Title Page of Humphrey Ridley's 1695 book '*The Anatomy of the Brain*'**. This test contains a comment on the 'tightness' of cerebral blood vessels, approximately 150 years before the experiments of the German scientists Ehrlich, Goldmann and Lewandowsky.

Some 150 years before the time considered the 'beginning' of brain barrier experiments, as conducted by Ehrlich and Goldmann, the first notions of specific protective nature of the brain were being described. Humphrey Ridley (1653-1708 [55]) a London physician distinguished himself by publishing a monograph titled '*The Anatomy of the Brain. Containing its Mechanism and Physiology; Together with some New Discoveries and Corrections of Ancient and Modern Authors Upon that Subject. To which is annex'd a particular Account of Animal Functions and Muscular Motion' *in 1695 ([56]; see recto title page in Figure 5). In it he described the importance of working with human subjects for dissection, enabling a proper understanding on the anatomical position of the intricacies and fine structure of the human form, as opposed to many earlier anatomists who worked mainly on 'lower' animals. Most important to Ridley's understanding of human anatomy was his use of not only observation, but also experimentation:

'... I have offer'd nothing but Matter of Fact, and have taken all possible care to avoid being impos'd upon my self [*sic*], by making Experiments in proportion to my Doubts. Some of them have been upon Subjects in their natural, some in their morbid [state], some upon those of Untimely Death; and on those last sometimes whist the natural Fluids remained in their proper Vessels, though after a preternatural manner occasion'd by Strangulation; sometimes when in the room thereof, other Bodies have been introduc'd by Injection, as Tinged Wax and Mercury, the first of which by its consistence chiefly, the other by its permanent nature and colour, contribute mightily towards bringing to view the most minute ramifications of Vessels, and secretes recesses of Nature.'

This description of his scientific methods is of interest to the history of the brain barriers as Ridley appears to be the first to describe the impermeability of the cerebral blood vasculature to a substance injected into the bloodstream:

'... they [the nerves] do rarely come to sight in any form at all, Wax being over gross a body to enter such minute Vessels as those are; whereas by an injection with Mercury I find scarce any Nerves but what hath some small ramifications of Blood-vessels in them.'

Though not fully understanding the nature of his finding, Ridley does understand its importance:

'... I shall therefore only take notice of such propagations of them [the blood vessels], as are either remarkable for magnitude, some curiosity of Structure, or useful design of Nature.'

These observations were made years before the widely-cited dye experiments of Ehrlich that are generally considered to be the origin of the concept of a "blood-brain barrier". It seems most unfortunate that Ridley's truly insightful description of the impermeability and importance of cerebral blood vessels is not quoted by historians of the medical sciences, or this particular field, possibly due to the non-nobility of Ridley's background and learning. It is known that Ridley entered a medical course at Merton College, Oxford, in 1671 where he 'played the fiddle as much as the book' [55]. He then left the University without a degree, but was later 'doctorated in physic [medicine]' at the University of Cambridge. Although forgotten to the depths of time, some have appreciated the additions Ridley made to the neurosciences, stating that his book '*The Anatomy of the Brain...'*:

'... shew [*sic*] him much above the ridiculous medical fiddle faddle of that age of ignorance and quackery.' [55]

Following the publications of Humphrey Ridley, little work was reported relating to the barriers of the brain until the notion that a stable internal environment in the body is important for normal physiological functioning was postulated by the French physiologist Claude Bernard (1813 - 1878 [84]). Bernard's '*millieu intérieur*' was explained by him as:

'The constancy of the internal environment is the condition for a free and independent life'

Although imposed on a whole body system, the description of the importance of homeostasis by Bernard is important, as up until this time there had been no descriptions of the significance of the controlled internal environment of the human (or animal) body.

Not long after, in 1885, the German scientist and Nobel Laureate, Paul Ehrlich (1854 - 1915 [85]) injected dyes into the peritoneum of animals. In these experiments, practically all of the organs of the animal were stained, except for the brain and spinal cord [86]. Although Ehrlich himself described the observation of the lack of staining, his thoughts were not as insightful as Ridley's in 1695 since he presumed, as mentioned above, that the difference was due to the different binding affinities of different tissues to different dyes.

**Figure 6:**
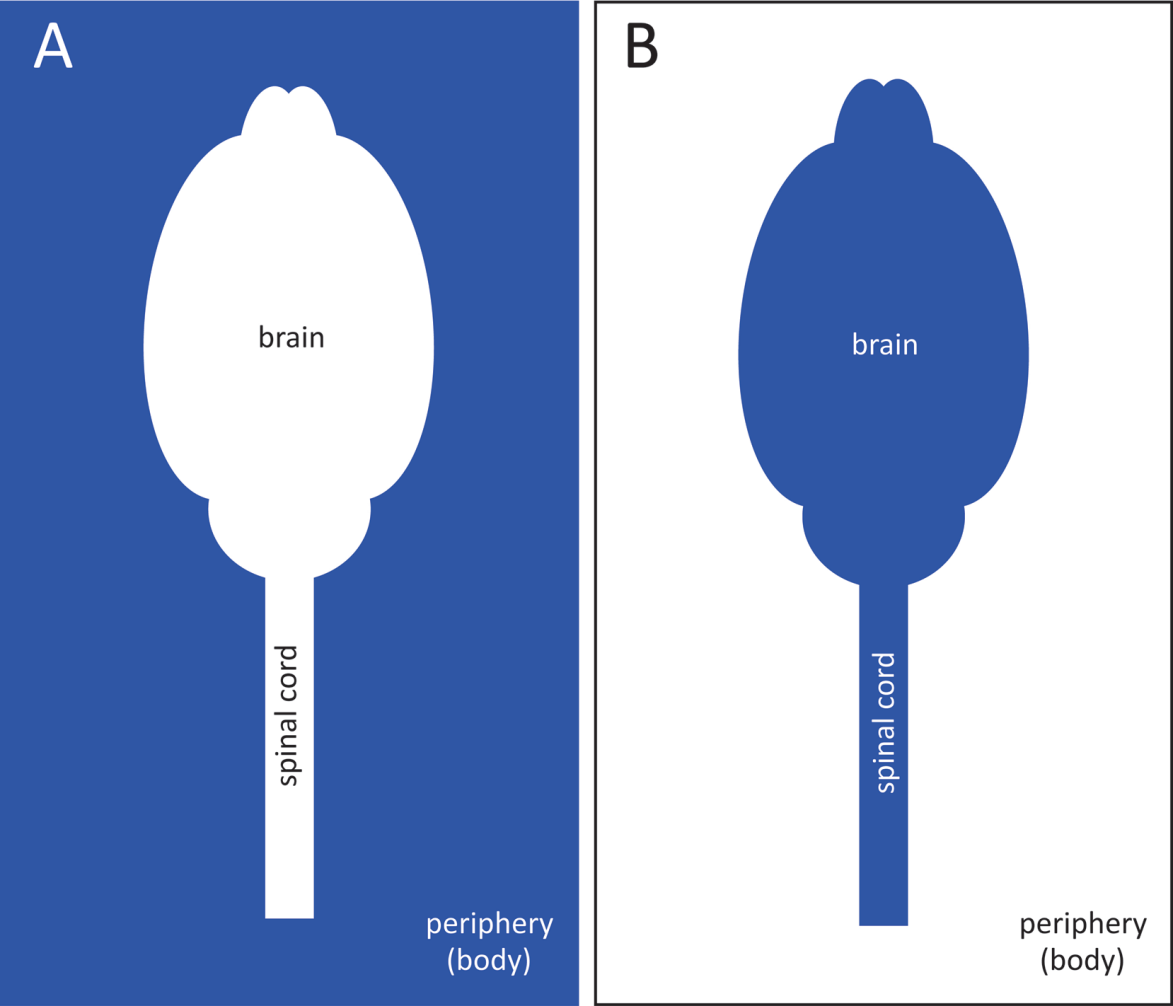
**Illustration of early brain barrier experiments by Ehrlich and Goldmann**. These early experiments elegantly demonstrated the compartmentalisation between the central nervous system (brain and spinal cord) and the peripheral organs. **A**. Trypan blue is delivered peripherally [86,88]. The dye does not penetrate any organs of the central nervous system, which both researchers suggested was due to the central nervous system having a lower affinity than other tissues. **B**. Trypan blue is injected into the brain [12]. The brain and spinal cord were stained, while the peripheral organs were not.

One of Ehrlich's students, Edwin Goldmann (1862 - 1913 [87]) was integral to helping form the early concept of the brain barriers when he completed the converse of Ehrlich's original dye experiment by injecting the dye trypan blue (MW 960Da) directly into the CSF of the brain. In these experiments, Goldmann found that the brain itself was stained, but the body of the animal was not. After intravenous injection of the same dye, Goldmann described no staining in the nervous system, aside from the presence of the reaction product in the choroid plexus and pineal gland [88]. These experiments clearly demonstrated the existence of a compartmentalization between the brain and the rest of the body. Goldmann also hypothesized that the vehicle for substance transport to the brain was the CSF, which was gaining access to the brain tissue via the choroid plexuses. An outline of these experiments is provided in Figure 6.

The existence of a physical barrier at the level of the cerebral blood vessels was first hypothesized by Max Lewandowsky (1876 - 1918 [89]) in his 1900 publication '*Zur Lehre der Zerebrospinalflussigkeit*'. In these experiments Lewandowsky injected either cholic acids or sodium ferrocyanide, and described that they had no pharmacological effects on the nervous system, whereas symptoms did occur after injection directly into the ventricles of the brain [90]. He concluded that:

'... the walls of cerebral capillaries hinder the transit of certain compounds and not for others.'

Although encountering much scepticism, including from Ehrlich, it was Lewandowsky who coined the term 'bluthirnschranke' or 'blood brain barrier'.

At the same time the understanding of the brain barriers was advancing in Germany, two English scientists Charles Roy (1854 - 1897 [91]) and Charles Sherrington (1857 - 1952 [92]) noted that the brain contained some way of ensuring the vascular supply was segregated from the neuronal tissue. They comment:

'... the brain possesses an intrinsic mechanism by which its vascular supply can be varied locally in correspondence with local variations of functional activity.' [93]

They also investigated the movement of a range of substances into the brain, across the brain barriers. Though apparently unaware of the importance of their findings for understanding of the concept of the blood brain barrier, they did report that many lipid-soluble molecules such as morphine and caffeine can cross into the brain, while other, lipid-insoluble molecules, cannot [93].

Further support for the presence of the blood-brain barrier came in 1921 from the Russian neurophysiologist, Lina Stern (1878 - 1968 [94]). She was the first woman to receive the rank of Professor at the University of Geneva, and was also an avid supporter and then member of the Jewish Anti-Fascist Committee in the USSR, which was wiped out in 1949 when all members were sentenced to death - it was only Stern who survived to continue her work [94]. During her scientific career, she was mystified why certain medicines administered to the blood stream did not enter the central nervous system. Intravenous injections of anti-tetanus medicine failed to check tetanus once the poison entered the central nervous system, leading her to conclude that there must be a barrier that protects the nervous system from toxins and germs, which she called the 'hematoencephalic barrier'. It has been noted that Stern and colleagues regarded the brain barrier as an absolute barrier rather than a restraint that slowed the passage of solutes between the blood and the brain - a fact that Hugh Davson suggested is an intellectual inadequacy of the model [95].

There was still much controversy surrounding the actual presence of the brain barriers, even up until the 1950s, when the lack of penetration of these dyes was suggested to be most likely due to their tight binding to plasma protein, particularly albumin, in the blood [96]. The advent of the electron microscope enabled pioneering and elegant experiments that looked at the transfer of HRP. Reese and Karnovsky [97] showed, for the first time, that in the mouse cerebral capillaries, HRP was able to enter the interendothelial spaces up to, but not beyond, the first tight junction between adjacent capillary endothelial cells. This result was repeated with ever increasingly smaller molecules such as microperoxidase (MW 1900 Da and ionic radius 2 nm [98]) and lanthanum ion (MW 139 Da ionic radius 0.115 nm [68]). Around the same time, Reese and Karnovsky showed that it was not the astrocytic endfeet or the basement membrane that was forming the barrier, but the endothelium itself [97].

As the presence of tight junctions between cerebral endothelial cells became known, the fact that they were more functionally tight than, for example, the junctions found in blood vessels of skeletal muscle, was further investigated. Using freeze-fracture electron microscopy it was shown that the tight junctions between endothelial cells of both the cerebral vascular (capillaries and venules) were arranged in about 6-8 parallel strands [62,99]. This structure caused 'tightness' of the barrier for molecules down to around 10-15Å and a very high transendothelial electrical resistance [100,101]. The results on the movement of electron dense tracers at the site of the blood-brain barrier were supplemented with the observation that the choroid plexus was also able to halt the movement of HRP from the blood to the CSF [68].

## Conclusions

As stated in the first article published in this journal [102] - the collective study of the barriers of the brain is important for the understanding of health and disease in the broadest sense. The close homeostatic control of the internal milieu of the central nervous system: the cerebrospinal fluid and neurons, plays a vital role in normal and abnormal brain function. Dysfunctional brain barriers contribute heavily to the pathology of neurological conditions ranging from trauma, to diseases of neuronal development and degeneration. In addition, a proper understanding of these protective barriers has the potential to prove important for the production of pharmacological deliverables that may be able to help ameliorate neurological diseases.

## Competing interests

The authors declare that they have no competing interests.

## Authors' contributions

SAL is sole author and has read and approved the final version of the manuscript.
